# Coupling Relationship Between Soil Properties and Plant Diversity Under Different Ecological Restoration Patterns in the Abandoned Coal Mine Area of Southern China

**DOI:** 10.1002/ece3.70686

**Published:** 2024-12-17

**Authors:** Hao Li, Wenbo Chen, Jintai Li, Cheng Zhang, Haifen Liang

**Affiliations:** ^1^ School of Surveying and Geoinformation Engineering East China University of Technology Nanchang China; ^2^ Jiangxi Bureau of Geology Energy Geology Brigade Nanchang China; ^3^ College of Land Resource and Environment Jiangxi Agricultural University Nanchang China; ^4^ Jiangxi Key Laboratory of Watershed Ecological Process and Information East China University of Technology Nanchang China; ^5^ Nanchang Key Laboratory of Landscape Process and Territorial Spatial Ecological Restoration East China University of Technology Nanchang China; ^6^ National Key Laboratory of Uranium Resource Exploration‐Mining and Nuclear Remote Sensing East China University of Technology Nanchang China

**Keywords:** abandoned coal mine area, ecological restoration, plant diversity, soil properties, vegetation configuration

## Abstract

Understanding the effects of ecological restoration in abandoned coal mines on soil and plant is important to improve the knowledge of ecosystems evolution and facilitate taking appropriate ecological restoration management practices. This study aims to evaluate the coupling relationship between plant diversity and soil properties after ecological restoration in abandoned coal mine area. The plant diversity such as Margalef index (*M*), Simpson index (*H*), Shannon–Wiener index (*D*), and Pielou index (*J*), and soil properties such as soil pH, soil water content (SWC), soil bulk density (SBD), soil organic matter (SOM), available nitrogen (AN), and microbial biomass carbon (MBC) were investigated in four sites of different ecological restoration patterns, T and study the coupling relationship between them. The results indicated that: (1) the *Pinus massoniana* and *Schima superba* gardner and champ restoration (PSR) site had higher Shannon–Wiener index and Simpson index values than *Pinus massoniana* restoration (PR) site, and in herb layer, the plant diversity was significantly higher than in other layers; (2) in the PSR site, the soil properties were improved more notably than that of PR and nature restoration (NR) sites, and the plant diversity were also higher than PR site; (3) Clay, SOM, and MBC made a great contribution to the plant diversity. It was concluded that ecological restoration patterns had significant effects on soil nutrient content and plant diversity, and there exists evident coupling relationship between plant diversity and soil properties. This study has important effects of ecological restoration and management in abandoned coal mine area.

## Graphical abstract

This study evaluated the coupling relationship between plant diversity and soil properties after ecological restoration in abandoned coal mine area. The plant diversity of arbor, shrub, and herb layers, and the soil properties in different depth layers were investigated in four sites of different ecological restoration patterns. Ecological restoration patterns had significantly effect on plant diversity. There was significant differences in plant diversity and ecological restoration patterns.

Aphids host endosymbionts protect them against attacks from parasitoids, leading to potential reduction in the efficacy of biological control against these crop pests. We developed a Monte Carlo simulation model to examine changes in levels of endosymbiont infection in an insect population in the presence of parasitoids attacking them over several generations, and to quantify potential reductions in the efficacy of parasitoids in controlling host populations in biological control. Results suggest that longevity of parasitoids and the spatial aggregation of hosts likely play a major role in the dynamics of symbiont infection.

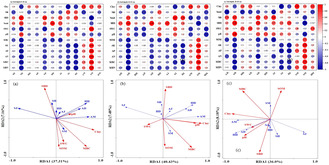

## 1 Introduction

Coal is one of the three primary energy resources in the world, and the exploited of coal accounts for one‐third of the world's energy consumption (Gao et al. 2021). China is the largest producer of coal, and coal is also the major energy resource in the nation's energy supply, such as power fuel and to generate electricity, and its dominance will continue for a long time (Ruan et al. 2022; Wu et al. 2020; Yuan et al. 2022). Coal mining activities cause environmental damage, such as landscape fragmentation, species loss, vegetation elimination and soil degradation (Babí Almenar et al. 2019; Lechner et al. 2016). Moreover, underground coal mining may cause land subsidence and produce large quantities of mine waste, having an irreversible damage to ecosystem development (Gomes, Mendes, and Costa 2011; Lechner et al. 2016). The ecological environment background conditions of coal mine areas are always very poor, due to coal excavation, coal washing and coal gangue disposal, seriously threatening the safety of people and property (Ahirwal and Maiti 2018). In China, coal resource utilization has recently increased rapidly due to the long‐term dependence of the economic development (Xie et al. 2023). The area destroyed by mining activities has increased to 120,000 km^2^ in 2020, and the number of abandoned coal mines was more than 12,000 (Wang, Huang, and Chen 2022). Furthermore, in 2022, abandoned mines account for 30.35% of the mine development area, and only 4.64% has been restored (Lyu, Yang, and Fang 2022). Therefore, the implementation of ecological restoration in abandoned coal mine area is especially urgent. Ecological restoration is a main measure to maintain the stability of the ecosystem, and how to conduct ecological restoration scientifically and effectively has been highly valued by many researches (Ismaeel and Ali 2020; Li et al. 2022). The United Nations General Assembly proclaimed a 10‐year plan on ecosystem restoration to facilitate the restoration of damaged ecosystems (UNEP 2019), and the “13th 5‐Year Plan (2016–2020)” in China has given priority to ecological restoration of mining areas.

Recently, many studies on ecological restoration of abandoned coal mine areas have concentrated ecological restoration measures to improve soil properties. Vegetation restoration plays an important role in improving soil quality and restoring other ecological services in abandoned coal mine area (Kaiser‐Bunbury et al. 2017; Pandey and Bauddh 2018). Furthermore, vegetation restoration is not only less costly and more environmentally friendly than physical and chemical restoration, but also bring esthetic value and produce social economic benefits (Manhães et al. 2018; Pathak, Agarwal, and Vimal Chandra 2020).

Revegetation can improve the soil structure and physicochemical properties (Finkenbein et al. 2013). Appropriate vegetation restoration projects can significantly improve soil nutrient and activity (Mahar et al. 2016; Yuan et al. 2018). It is well known that soil properties like soil particle composition, soil nutrient and soil water condition, are conducive to the maintenance of plant diversity (Gong et al. 2019). Some researches reported that soil nutrients, soil pH, soil water content (SWC), and soil bulk density (SBD) had significant effect on plant diversity (Damgaard et al. 2013). Soil pH can change soil enzyme activity and nutrient, thus affecting plant diversity (Cambrollé et al. 2014). SWC and SBD plays a key role in soil hydrological processes, and the improvement of which is beneficial to improving ecosystem productivity and plant diversity (Boluwade and Madramootoo 2016; Katherine et al. 2010). Soil organic matter (SOM) is significantly correlated with available nitrogen (AN) and available phosphorus (AP) (Chen et al. 2019; Liu et al. 2021). Previous researches reported that soil heterogeneity and nutrients was thought to improve diversity and spatial heterogeneity of plant communities (Schweiger et al. 2016). Meanwhile, vegetation restoration can improve soil nutrient availability, and improve ecosystem productivity (Bakker et al. 2019). However, the influential mechanisms of soil properties on plant diversity are complex, and few studies are available for it (Lü et al. 2019; Wu et al. 2019).

Plant diversity is one of the most important feature in biodiversity, which can describe the structural complexity of plant community (Bakker et al. 2019). Plant diversity can be measured through the metrics of Margalef index (*M*), Simpson index (*D*), Shannon–Wiener index (*H*), and Pielou index (*J*) (Bennett, Radford, and Haslem 2006). Current researches on the coupling relationship between soil properties and plant diversity always concentrate on forests rather than coal mine restoration areas. How soil properties affect plant diversity, and how they interact in different vegetation configuration remains to be studied. Therefore, the research of coupling relationship between soil properties and plant diversity under different ecological restoration patterns plays an important role in providing theoretical guidance for abandoned coal mine restoration.

In this study, we analyze the effects of different vegetation restoration patterns on plant diversity and soil properties, as well as the relationship between them in abandoned coal mine areas. The aims of this study are to: (1) evaluate the change trend of soil properties and plant diversity in abandoned coal mine areas under different vegetation restoration patterns, (2) discover the relationship between soil properties and plant diversity, (3) determine impacting factors of soil properties and plant diversity from plant community's point of view. This study is expected to better understand the ecological restoration process and provide a basis for the management practices.

## 2 Materials and Methods

### 2.1 Study Sites

**FIGURE 1:**
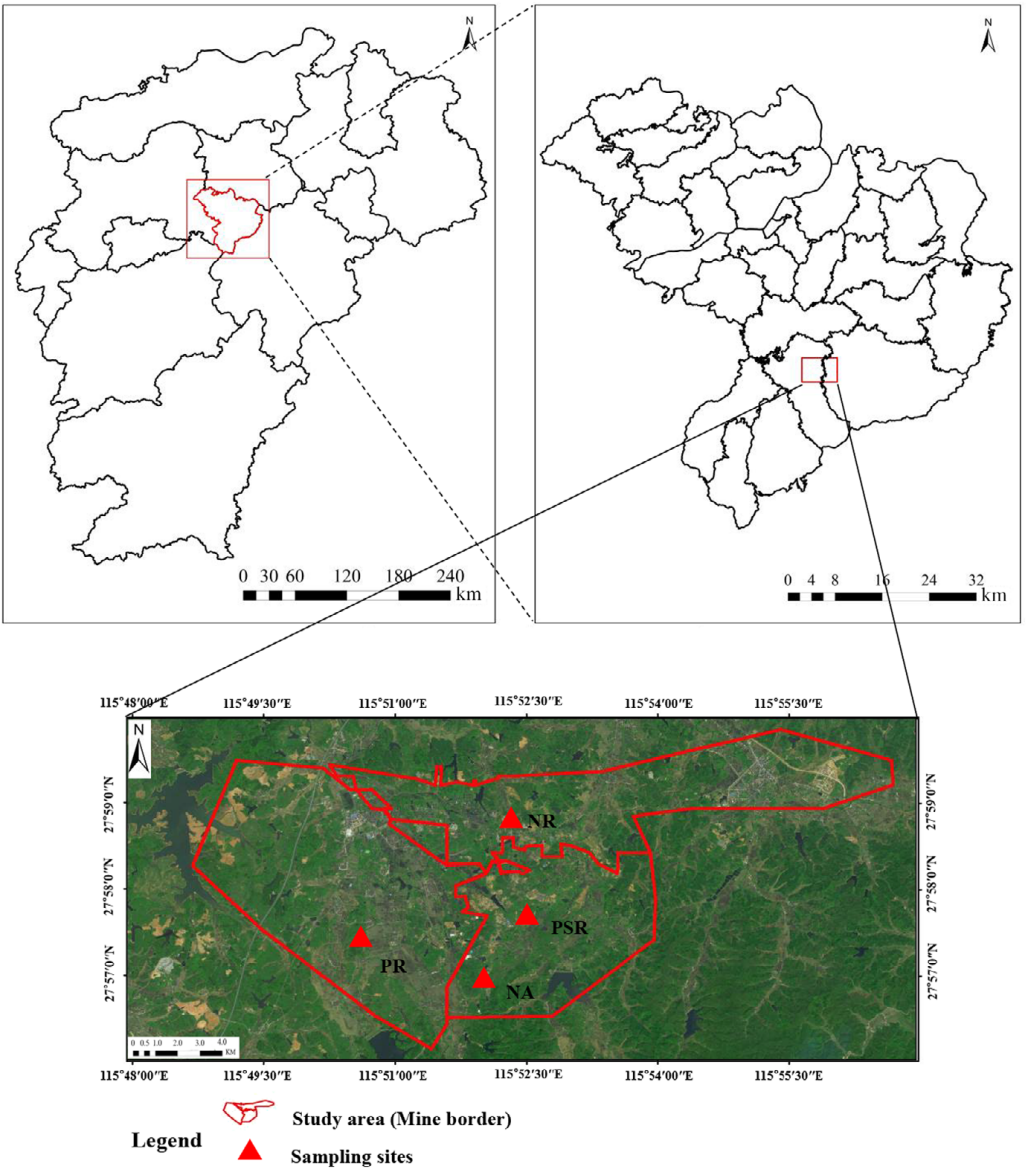
Location of study sites in the south of Fengcheng County, Jiangxi, China.

The study was carried out in the abandoned coal mines of Liushe, Shanxi and Longxi coal mine areas, covering a total area of 4276 hm^2^ (115°48′30″ *~* 115°57′30″ E, 27°56′00″ *~* 27°59′30″ N), located in Fengcheng county, Jiangxi province, China (Figure 1). The altitude ranges from 45 to 75 m, with an average of 60 m. The region has humid subtropical climate, with an average annual temperature of 15.3°C–17.7°C. More than 50% of the annual rainfall occurs from April to June, with an annual average rainfall of 1552.1 mm. Bedrocks are classified as phyllite and sandshale. Soils are classified as red earth, mostly medium and heavy loam.

The restoration process of closed coal mine areas includes two types of practices: active and passive. The active‐type procedures are based on human intervention, including physical restoration and phytoremediation, such as improvement of soil fertility and plantation of dominant species ( *Pinus massoniana* and *Schima superba* gardner and champ). The passive type is established upon natural regeneration processes. In the active restoration area, vegetation was planted at 3 × 3 m intervals, and about 940,000 trees were planted in 850 hm^2^ soil degradation area. After 3 years of management, the planted vegetation began to natural succession. At present, the study sites have formed a multi‐level and multi‐type vegetation structure of trees, shrubs, and herbs. And the plots were installed in 2010–2012, after the ecological restoration of the abandoned coal mine. Before the experiment was installed, study area has serious geological environmental problems caused by coal mine activity, such as deformation of the surface morphology, declining water levels, soil erosion and deterioration of soil and plant.

*Pinus massoniana* and *Schima superba* gardner and champ are the two main plantation tree species in south China, which have the advantages of fast growth and wide use, playing a vital role in protecting the ecological environment and maintaining the balance of the ecosystem. *Pinus massoniana* is a species with deep roots, and growth generally requires higher light and accumulated temperature (Cheng, Hong, and Wu 2006). Compared with other species, *Pinus massoniana* has lower light energy utilization efficiency, more sensitive reaction to heterogeneous nutrient environment, and higher nutrient absorption efficiency. The ecological restoration of study area is dominated by planting *Pinus massoniana* monoculture forest and *Pinus massoniana* × *Schima superba* gardner and champ mixed forest, with a close hillsides to facilitate afforestation and natural succession. Therefore, this area is characterized by a relatively simple community structure, and the main tree species in the arborous layer are *Pinus massoniana* and *Schima superba* gardner and champ. The shrubs in the study area are mainly *Osmanthus fragrans* var.*semperflorens*, *Photinia* × *fraseri* Dress, *Camellia japonica* L., and *Lagerstroemia indica* L., and the herbs are *Cynodon dactylon* L., *Setaria viridis* L., *Dendranthema indicum*, and *Poa annua* L.

### 2.2 Sites Selection, Plant Investigation and Soil Sampling

A series of ecological restoration plantations was established, including *Pinus massoniana* restoration, *Pinus massoniana* and *Schima superba* gardner and champ mixed restoration, nature restoration. Considering similar soil climate conditions, and land use histories, historical record databases were selected as research samples, providing different vegetation restoration patterns of abandoned coal mine ecological restoration. Four typical sites of different ecological restoration patterns were selected: *Pinus massoniana* restoration (PR), *Pinus massoniana* and *Schima superba* gardner and champ restoration (PSR), nature restoration (NR), and a NA (nature undisturbed area) as the reference control condition (Table 1). For each ecological restoration pattern, considering both the location and slope, we choose two sample sites in the study area, and randomly established five plots on land that had relatively homogeneous surface coverage in each site. The latitude, longitude, altitude and dominant species were recorded in each study sites. We made ground vegetation investigation to collect data on plant diversity in June 2022. Since the investigation sites were depended on the plant community size, five 10 × 10 m quadrat were selected in each vegetation ecological restoration site as arbor layer. Species name, quantities of trees, height, diameter of breast height (DBH) and crown breadth were recorded. Two 5 × 5 m quadrat were mechanically arranged as shrub layer squares in each arbor quadrat, and one 1 × 1 m herb layer quadrat was set in the center of each shrub quadrat (totaling 40 tree plots, each 10 × 10 m, 80 shrub plots, each 5 × 5 m, and 80 herb plots, each 1 × 1 m). The species name, average coverage, abundances and average height of each specie were recorded in shrub layer and herb layer, respectively.

**TABLE 1:** Information on the five study sites.

Types	Altitude (m)	Longitude	Latitude	Restoration years	Dominant species	Plots
PR	68.23	115°50′36″	27°57′21″	10	*Pinus massoniana* , *Camellia japonica* L., *Photinia × fraseri* Dress	Arbor: 10
						Shrub: 20
						Herb: 20
PSR	75.44	115°52′35″	27°57′38″	10	*Pinus massoniana* , *Schima superba* gardner and champ, *Photinia × fraseri* Dress	Arbor: 10
						Shrub: 20
						Herb: 20
NR	65.37	115°52′16″	27°58′48″	10	*Pinus massoniana* , *Cunninghamia lanceolata* , *Camellia japonica* L., *Pyracantha fortuneana*	Arbor: 10
						Shrub: 20
						Herb: 20
NA	46.54	115°52′15″	27°56′42″	> 20	*Cunninghamia lanceolata* , *Schima superba* gardner and champ, *Osmanthus fragrans* var. *Semperflorens*, *Photinia × fraseri* Dress	Arbor: 10
						Shrub: 20
						Herb: 20

Considering the characteristics of soil properties in the top layer, each soil profile was sampled for every 10 cm by auger from three layers: 0–10 cm, 10–20 cm, and 20–30 cm. Soil samples were collected along an S‐shaped pattern from each study site, and a total of 100 soil samples were collected for soil properties determination. At each sampling site, approximately 0.5–1 kg of soil sample was selected according to the quartet method after removing plant roots, stones, weeds and litter. After air‐drying, the collected soil samples were crushed, and passed through a sieve (2 and 1 mm). Finally, soil physical and chemical properties, such as soil particle composition, pH, soil organic matter, and soil available nutrient… were determined.

### 2.3 Plant Diversity Analysis and Soil Properties Measurement

The importance value (IV) and plant diversity index of different plant layers were calculated through the plant investigation data. IV is an essential species diversity index and the IV value can directly indicate the relative importance of plant species in a community (Zhang et al. 2011). In this study, the plant diversity index *H*, *D*, *J*, and *M* were calculated to describe the plant diversity in different ecological restoration patterns area (Kumar et al. 2015; Zhou et al. 2016). The calculation methods were as follows:

(1) $$\text{Important value}\;\left(\mathrm{IV}\right)=\left(\text{Relative density}+\text{Relative frequency}+\text{Relative coverage}\right)/3$$

where the relative density is the density of a species/sum of the densities of all species; the relative frequency is the frequency of a species/sum of the frequencies of all species; the relative coverage is the plant coverage of a species/sum of plant coverage of all species.

Shannon–Wiener diversity index (*H*):

(2) $$H=-\sum_{i=1}^{S}\mathrm{Pi}\ln\mathrm{Pi}$$

Simpson diversity index (*D*):

(3) $$D=1-\sum_{i=1}^{S}{\mathrm{Pi}}^{2}$$

Pielou evenness index (*J*):

(4) J=HlnS

Margalef richness index (*M*):

(5) M=S−1lnN

where *P*_i_ is the ratio of the number of individuals, *i* is the base of the logarithm, *P*_i_ = *n*_i_/*n* and *i* = 1, 2, 3,…*n*_i_, of species *i* in the sample to the total number of individuals, *n*, of the species in the sample, *S* is the number of species in the sample quadrat, and *N* is the number of all plants in the sample quadrat.

SBD and SWC were determined separately by the stainless‐steel cylinder and gravimetric method, and the soil mechanical composition (clay/silt/sand) was measured using the dry sieving and aqueous suspension settling time method. Soil pH was measured using a water–soil ratio of 2.5:1.0 and the potentiometric method. SOM was measured by the K_2_Cr_2_O_7_ oxidation‐external heating method. The contents of soil AN was measured by NaOH hydrolysis proliferation by the alkalihydrolysis method, AP was measured using the molybdenum‐antimony colorimetric method and AK was measured by flame atomic absorption spectrophotometry method (Liao et al. 2014). The contents of soil microbial biomass carbon (MBC) and soil microbial biomass nitrogen (MBN) were determined by the chloroform fumigation extraction method (Dong et al. 2018).

### 2.4 Statistical Analysis

One‐way analysis of variance (ANOVA) was used to analyze the significance of the difference among soil properties and plant diversity indexes in different ecological restoration patterns at a significance level of *p* < 0.05, and Duncan post hoc multiple comparisons test (*p* < 0.05) was used to compare the means of soil properties and plant diversity indexes. Statistical analysis was performed using SPSS 26 (IBM SPSS Statistics 26). Pearson's correlation coefficient was used to quantify the relationship between soil properties and species diversity. The corresponding relationships between soil properties and species diversity were quantified and the ordination map was drawn using redundancy analysis (RDA) by the Canoco 5.0, and Origin 2019 was used to draw the graphs.

## 3 Results

### 3.1 Plant Diversity and Community Composition Under Different Ecological Restoration Patterns

The composition of the plant communities in each study area were shown in Table 2. In the sample plots, a total of 21 families, 29 genera, and 31 species were observed. A total of 24 species appeared in the NA site, and the dominant species were *Cunninghamia lanceolata* , *Osmanthus fragrans* var. *Semperflorens*, and *Setaria viridis* L. In the PR site, the number of plant species was 19, *Pinus massoniana* , *Photinia × fraseri* Dress *and* *Cynodon dactylon* L. were dominant species. There were 24 species in the PSR site, *Pinus massoniana* and *Schima superba* gardner and champ were revegetation plant species in arbor layer, the dominant species in shrub and herd layers were *Photinia × fraseri* Dress and *Cynodon dactylon* L. In the NR site, the number of plant species was 27, and the dominant species were *Pinus massoniana* , *Cunninghamia lanceolata* , *Osmanthus fragrans* var. *Semperflorens*, *Camellia japonica* L., and *Cynodon dactylon* L.

**TABLE 2:** The composition of plant communities under different ecological restoration patterns.

Layer	Family	Genus	Species	Importance value			
				PR	PSR	NR	NA
Arbor layer	Pinaceae	Pinus	*Pinus massoniana*	0.66	0.31	0.17	0.11
	Pinaceae	Pseudolarix	*Pseudolarix amabilis*	0.1	0.13	0.13	0.12
	Cupressaceae	Sabina mill.	*Sabina chinensis*	0.05	0.06	0.12	—
	Taxodiaceae	Cunninghamia	*Cunninghamia lanceolata*	0.12	0.12	0.21	0.18
	Salicaceae	Salix	*Salix matsudana*	0.04	—	0.08	0.10
	Lauraceae	Cinnamomum	*Cinnamomum camphora*	—	0.05	0.10	0.07
	Scrophulariaceae	Paulownia	*Paulownia*	—	0.06	—	0.06
	Leguminosae sp.	Robinia L.	*Robinia pseudoacacia* L.	0.03	—	0.05	0.08
	Elaeocarpaceae	Elaeocarpus	*Elaeocarpus decipiens*	—	—	0.03	0.07
	Theaceae	Schima reinw	*Schima superba* gardner and champ	—	0.27	—	0.21
Shrub layer	*Osmanthus fragrans*	Osmanthus	*Osmanthus fragrans* var. *semperflorens*	0.14	0.06	0.12	0.32
	Rosaceae	Photinia Lindl	*Photinia × fraseri* Dress	0.25	0.23	0.08	0.21
	*Osmanthus fragrans*	Osmanthus Lour	*Osmanthus fragrans* cv.*tbubergii*	0.1	0.12	0.15	—
	Theaceae Mirb.	Camellia L.	*Camellia japonica* L.	0.37	0.16	0.23	0.18
	Rosaceae	Rose L.	*Rosa chinensis*	—	0.13	0.06	—
	Rosaceae	Pyracantha	*Pyracantha fortuneana*	0.06	0.15	0.17	0.14
	Theaceae Mirb.	Camellia L.	*Camellia oleifera abel*.	0.08	—	0.05	—
	LYTHRACEAE	Lagerstroemia L.	*Lagerstroemia indica* L.	—	0.12	0.08	0.16
	Malvaceae	Hibiscus L.	*Hibiscus mutabilis* L.	—	0.03	0.06	—
Herb layer	Gramineae	*Cynodon dactylon*	*Cynodon dactylon* L.	0.23	0.27	0.13	0.05
	Gramineae	Setaria beauv.	*Setaria viridis* L.	0.12	0.09	0.06	0.19
	Asteraceae	Dendranthema	*Dendranthema indicum*	0.08	0.11	0.10	0.22
	Poaceae	Poa L.	*Poa annua* L.	0.21	0.23	0.11	0.15
	Compositae	Artemisia	*Artemisia hedinii*	—	0.04	0.08	0.10
	Leguminosae sp.	Trifolium	*Clover*	0.16	0.11	0.16	—
	Poaceae	Miscanthus	*Miscanthus*	0.05	0.04	0.10	0.17
	Poaceae	Lolium	*Lolium perenne* L.	—	0.06	0.06	0.03
	Poaceae	Zoysia	*Zoysia japonica* *Steud*	—	—	0.08	0.02
	Poaceae	Buchloe engelm.	*Buchloe dactyloides*	—	0.05	—	0.05
	Poaceae	Eremochloa Buse	*Eremochloa ophiuroides*	0.07	—	0.07	—
	Poaceae	Zoysia	*Zoysia pacifica* *goudswaard*	0.08	—	0.05	0.02

**FIGURE 2:**
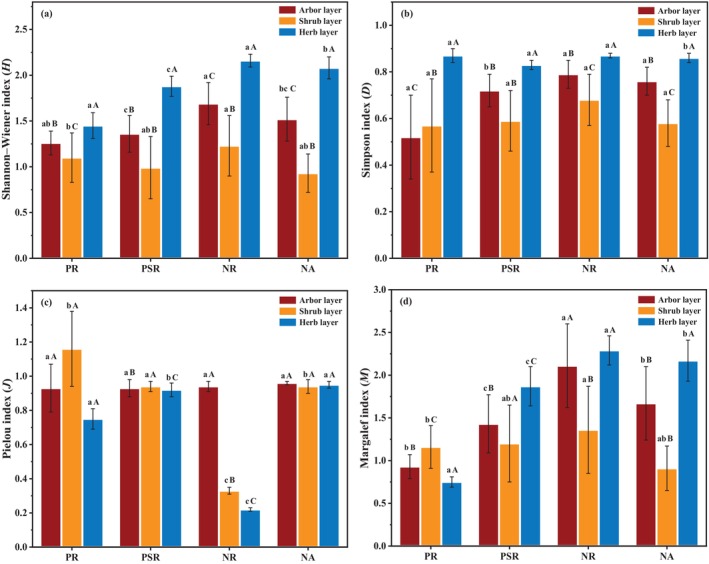
Diversity indexes of different ecological restoration patterns in the four study sites; (a) Shannon–Wiener index (*D*), (b) Simpson index (*H*), (c) Pielou index (*J*), (d) Margalef index (*M*). Different lowercase letters indicate significant difference under ecological restoration years (one‐way ANOVA, *p* < 0.05). Different uppercase letters indicate significant difference among different soil depths at the same sites (one‐way ANOVA, *p* < 0.05).

The plant diversity index *H* (Figure 2a) and *D* (Figure 2b) values did not differ significantly among the four study sites. The NR site had higher *D* and *H* values than PR site and PSR site, and the order of them was: NR > NA > PSR > PR. The plant diversity index *J* values had no significant differences among the four study sites (Figure 2c). It reached the highest in the PR site shrub layer, with the lowest value observed in the NA site herb layer. As it was seen in Figure 2d, the plant diversity index *M* had significant difference (*p* < 0.05) in different ecological restoration patterns. The shrub layer showed the lowest *M* value in PSR, NR and NA site, and the order of them was: NR > NA > PSR Overall, the plant diversity was slightly higher in the NR and NA site than those in PR and PSR sites. The results indicated that revegetation restoration community led to lower plant diversity than natural succession community did.

### 3.2 Soil Properties Under Different Ecological Restoration Patterns

**FIGURE 3:**
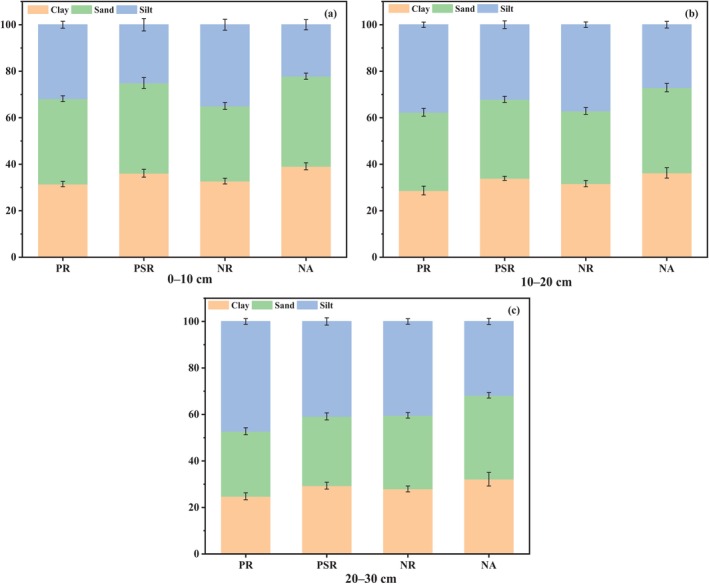
Mechanical composition (%) for different ecological restoration patterns at different study sites. (a) 0–10 cm soil layer, (b) 10–20 cm soil layer, (c) 20–30 cm soil layer.

**FIGURE 4:**
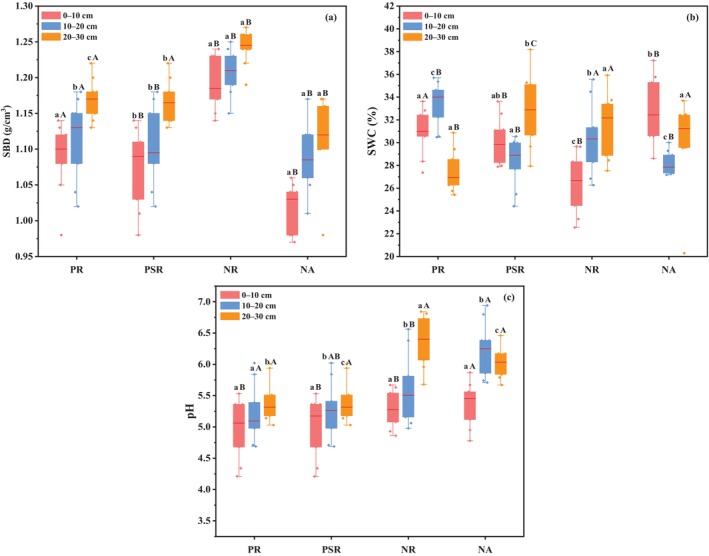
Soil physical indicators of different ecological restoration patterns in the four study sites on different soil layers. (a) SBD; (b) SWC; (c) pH.

Figure 3 showed the soil mechanical composition for different ecological restoration patterns. The PSR site and the NA site had a similar soil texture, which were significantly better (*p* < 0.05) than other ecological restoration patterns. The results indicated that when the time of the mixed vegetation restoration was more than 10 years, soil mechanical composition was close to the nature undisturbed area. The clay and sand contents increased slowly with the increasing of soil depth, but the silt content had an opposite trend. In Figure 4a, SBD was shown significantly lower (*p* < 0.05) in the PSR site than that in the PR and NR sites. Moreover, with the increase of soil depth, the SBD values showed an upward trend in all the four study sites. In Figure 4b, NA and PR sites were seen higher SWC value than the other study sites on the 0–10 soil layer. PR and PSR sites were seen lower pH value than the other study sites (Figure 4c). The results indicated that PSR site had better physical properties.

**FIGURE 5:**
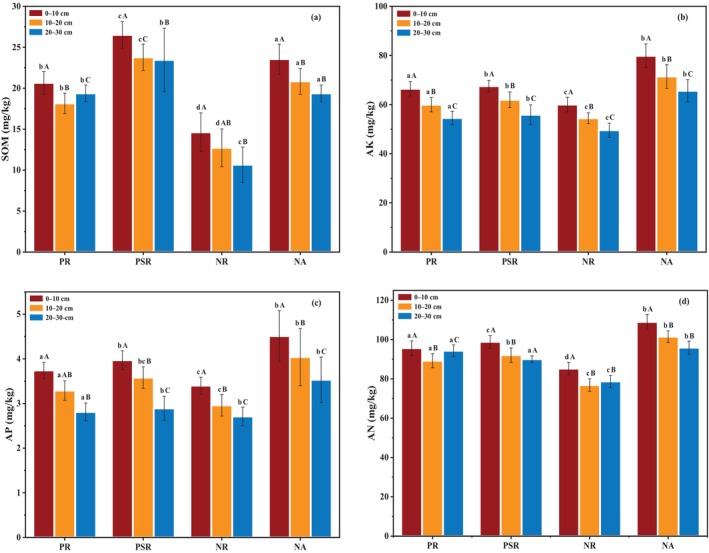
Soil chemical indicators of different ecological restoration patterns in the four study sites on different soil layers. (a) SOM; (b) AK; (c) AP; (d) AN.

Figure 5 showed the effects of different ecological restoration patterns on soil chemical properties. In Figure 5a, PSR site was significantly higher (*p* < 0.05) than that of the other study sites and exhibited the highest SOM value. Additionally, the SOM had a decrease trend with the increase of soil depth except PR site. As it was seen in Figure 5b, AK showed significant difference in PR, PSR and NR sites, and PSR site was significantly higher (*p* < 0.05) than that of PR and NR sites. Meanwhile, similar to SOM, AK value decreased with the increasing of soil depth. Similar to AK, PSR site was seen higher AP value than that of PR and NR sites (Figure 5c). In Figure 5d, AN showed significant difference in the four study sites, with the highest value observed in NA site and the lowest value in NR site. The results indicated that PSR site can significantly improve the chemical properties.

**FIGURE 6:**
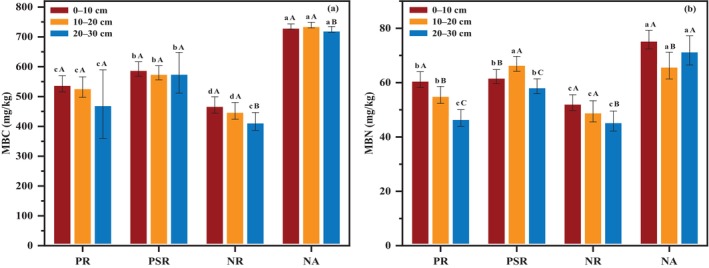
Soil microbial properties of different ecological restoration patterns in the four study sites on different soil layers. (a) MBC and (b) MBN.

Soil microbial properties of different ecological restoration patterns were showed in Figure 6. The MBC in PR, PSR, NR, and NA sites had significant difference, and the MBC values in PR and PSR sites were significantly (*p* < 0.05) higher than that in NR site (Figure 6a). The MBC value decreased slowly with the increasing of soil depth. In Figure 6b, the MBN was significantly higher in PSR site than that in PR and NR sites, and the MBN value decreased as follows: NA > PSR > PR > NA.

### 3.3 Coupling Relationship Between Plant Diversity and Soil Properties

**FIGURE 7:**
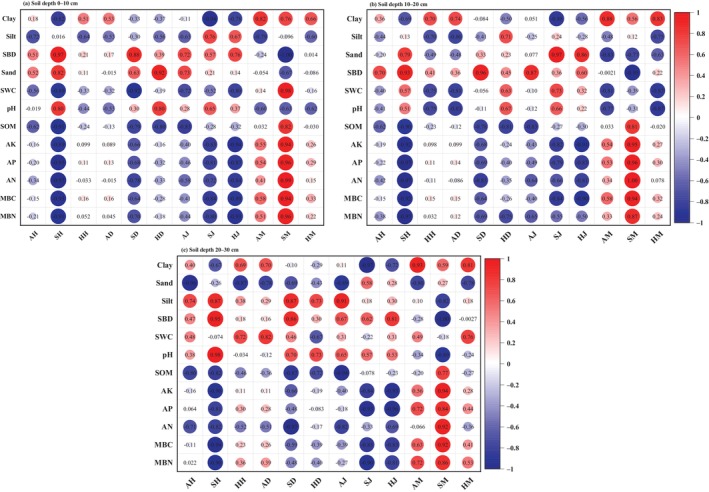
Correlation coefficients between soil properties and plant diversity indexes of different ecological restoration patterns sites at different soil depth layers. (a) 0–10 cm soil layer; (b) 10–20 cm soil layer; (c) 20–30 cm soil layer. AD, arbor layer Simpson index; AH, arbor layer Shannon–Wiener index; AJ, arbor layer Pielou index; AM, arbor layer Margalef index; HD, herb layer Simpson index; HH, herb layer Shannon–Wiener index; HJ, herb layer Pielou index; HM, herb layer Margalef index; SD, shrub layer Simpson index; SH, shrub layer Shannon–Wiener index; SJ, shrub layer Pielou index; SM, shrub layer Margalef index.

We calculated the correlation coefficients between plant diversity indexes (arbor layer Shannon–Wiener index (AH), shrub layer Shannon–Wiener index (SH), herb layer Shannon–Wiener index (HH), arbor layer Simpson index (AD), shrub layer Simpson index (SD), herb layer Simpson index (HD), arbor layer Pielou index (AJ), shrub layer Pielou index (SJ), herb layer Pielou index (HJ), arbor layer Margalef index (AM), shrub layer Margalef index (SM), herb layer Margalef index (HM)) and soil properties in different soil depths to reveal the effects of plant diversity on soil properties (Figure 7). The plant diversity *H* and *D* and evenness index *J* had a negative relationship with soil chemical properties and soil microbial properties (e.g., SOM, AK, AP, MBC…), and had a positive relationship with clay, SBD and SWC. Meanwhile, the correlation coefficient had a decreased trend with the increase of soil depth. Especially, on the 0–10 cm soil layer, the correlation among SH, SD, SJ, and SM, and clay, SWC and MBC became more significant. It indicated that plant diversity in shrub layer significantly affected the topsoil properties. While the correlation between arbor layer plant diversity and soil properties became more significant in 20–30 cm soil layer. This result suggested that the plant diversity indexes in arbor layer had great contribution to the deep soil properties. So we can draw the conclusion that the plant diversity in arbor layer, shrub layer, and herb layer had a deep impact on soil properties.

**FIGURE 8:**
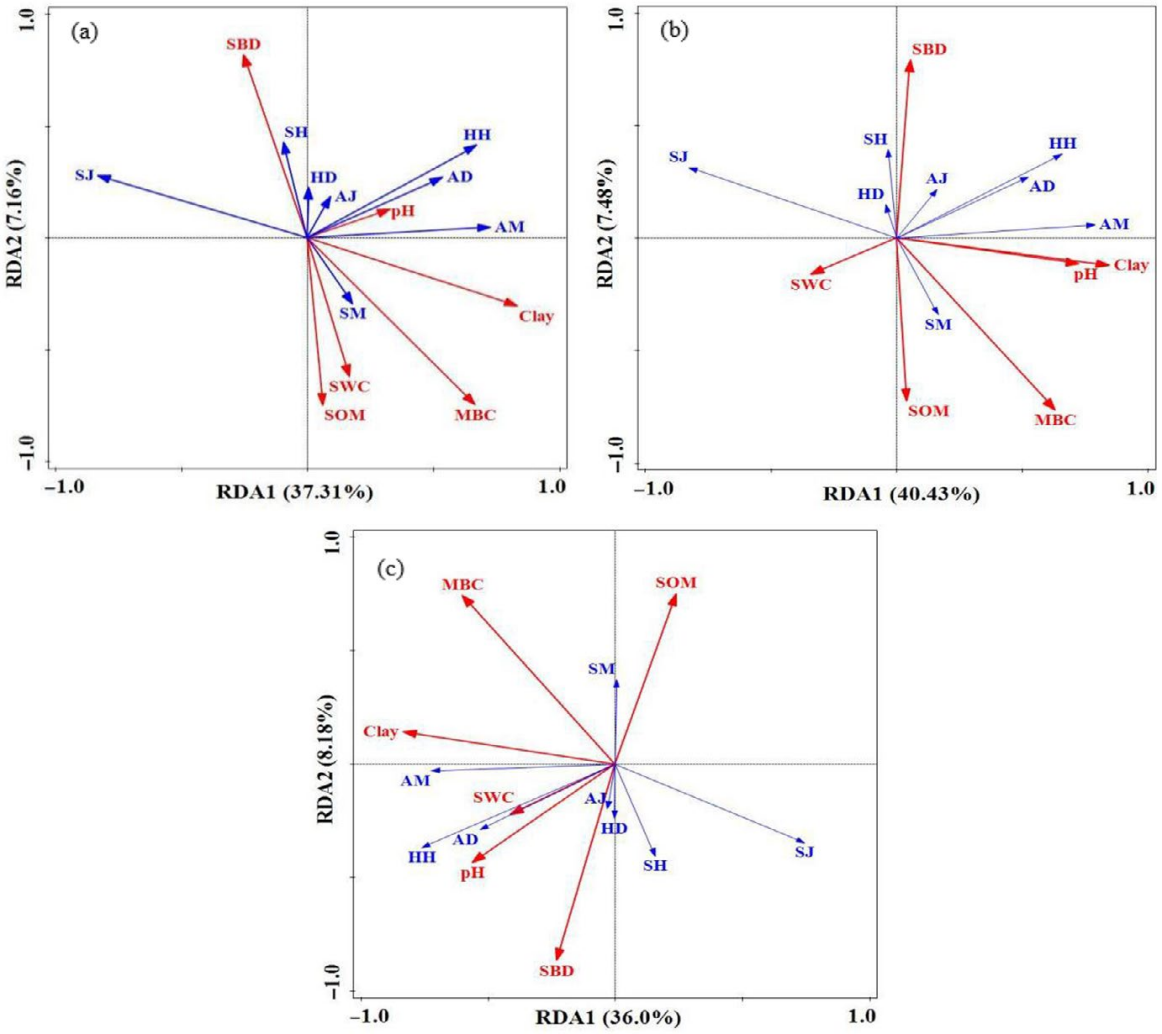
Redundancy analysis of soil l properties and plant diversity indexes of tree, shrub and herb layers at different soil depth layers. (a) 0–10 cm soil layer; (b) 10–20 cm soil layer; (c) 20–30 cm soil layer.

In order to deeply discover how plant diversity and soil property affect plant community, RDA model was used to analyze the corresponding relationships of soil properties in different soil depth layers and plant diversity of all layers (Figure 8, and Table 3). The results indicated that species composition was significantly affected soil properties on different soil depth layers. Clay, SOM, and MBN made a great contribution to the plant diversity. They were the most important explanatory variables in the RDA developed to explain plant diversity. In particular, SOM had substantially greater explanatory power for plant diversity than other soil properties, indicating that soil nutrient, especially soil organic carbon can explain plant diversity patterns better.

**TABLE 3:** Soil properties explanatory variables and contributions to the vegetation composition.

Index	Soil depth (cm)	Explains %	Contribution %	pseudo‐F	*p*	Variance inflation factor (VIF)
Sand	0–10	23.30	40.50	11.60	0.002	2.69
	10–20	1.90	3.10	1.30	0.26	5.39
	20–30	1.90	3.20	1.30	0.24	4.28
SOM	0–10	12.50	21.70	7.20	0.002	2.78
	10–20	15.50	26.20	9.60	0.002	5.74
	20–30	19.60	32.80	12.30	0.002	4.20
Clay	0–10	8.60	15.00	5.60	0.004	3.82
	10–20	24.50	41.30	12.30	0.002	3.64
	20–30	2.70	4.50	1.80	0.11	3.12
MBN	0–10	4.80	8.40	3.30	0.014	8.75
	10–20	0.90	1.50	0.60	0.69	6.88
	20–30	21.40	35.80	10.30	0.002	7.48
AN	0–10	2.40	4.20	1.70	0.15	7.22
	10–20	2.00	3.50	1.40	0.19	8.53
	20–30	0.90	1.50	0.60	0.69	4.24
SWC	0–10	1.20	2.10	0.90	0.49	2.33
	10–20	0.40	0.80	0.30	0.92	2.80
	20–30	5.40	9.10	3.60	0.014	1.89
Silt	0–10	1.10	2.00	0.80	0.54	1.83
	10–20	0.90	1.40	0.60	0.73	2.26
	20–30	2.00	3.30	1.40	0.18	2.85
MBC	0–10	1.00	1.80	0.70	0.58	16.32
	10–20	6.10	10.30	4.10	0.004	10.98
	20–30	0.80	1.30	0.50	0.75	4.02
pH	0–10	0.70	1.20	0.50	0.83	1.78
	10–20	3.60	6.00	2.50	0.044	1.76
	20–30	1.10	1.90	0.80	0.52	2.60
AP	0–10	0.70	1.20	0.50	0.84	3.22
	10–20	1.20	2.00	0.90	0.51	3.06
	20–30	1.70	2.90	1.20	0.29	2.69
SBD	0–10	0.70	1.20	0.50	0.83	4.69
	10–20	1.70	2.90	1.20	0.31	2.23
	20–30	1.90	3.20	1.30	0.23	5.48
AK	0–10	0.40	0.60	0.20	0.97	7.52
	10–20	0.60	1.00	0.40	0.86	5.93
	20–30	0.30	0.50	0.20	0.98	3.85

## 4 Discussion

### 4.1 Plant Diversity in Different Ecological Restoration Patterns

Plant diversity index *H*, *D*, *J*, and *M* could quantification the plant community composition and diversity (Zhu et al. 2017). In this study, the plant diversity index *H*, *D*, *J*, and *M* in the different ecological restoration patterns were gradually close to the NA site, indicating that revegetation is beneficial to reestablish plant community and restore plant diversity in abandoned mines, which was similar to other studies (Zhang et al. 2023). However, high plant diversity index did not mean stable community (Wang et al. 2019). Compared to the *M*, *D* and *H* (Jiang et al. 2022; Wang et al. 2019), the evenness index *J* in the plant communities can also help maintain the community stability (Zhang et al. 2023). In this study, the plant diversity index *D* and *H* and richness index *M* were higher in the NR site. The PSR site showed similar plant diversity index with NA site and lower than NR site (Figure 2), which indicated that although the plant diversity was lower, the community structure was more stable (Thomas, Amy, and Tara 2007).

In this study, the plant diversity index (Figure 2) in PSR site was much higher than the PR site, indicating that ecological restoration patterns of mixed vegetation was more effective in promoting the revegetation process of plant diversity. The results also showed that for the abandoned coal mine area vegetation ecological restoration, planting dominant plants mixed with other vegetation was a suitable measure to efficiently rebuild ecological functions. Therefore, identification of dominant plants in revegetation is of great importance for the species selection in abandoned coal mine area.

### 4.2 Soil Properties in Different Ecological Restoration Patterns

The vegetation restoration was an effective measurement in improvement of soil properties and had a significant effect in the ecological restoration of abandoned coal mine areas (Bi et al. 2021). Our results indicated that soil properties in most of study sites were significantly improved, indicating that soil nutrient content improved significantly with the process of vegetation restoration (Deng et al. 2018). In this study, significant change was also observed in soil mechanical composition. In PSR site, the clay and sand contents were higher than those in PR and NR sites (Figure 3), but the silt was lower, indicating that mixed vegetation restoration can improve soil particles and prevent the loss of soil nutrients (Gao and Huang 2020). SBD and SWC played an important role in soil development by affecting plant growth and nutrient utilization (Mora and Lázaro 2014; Salazar et al. 2009). PSR and NA sites showed lower SBD and higher SWC than other sites (Figure 4). The results were mainly due to the decay of roots generating persistent macropores in the soil, thereby improving the follow of water (Mitchell, Ellsworth, and Meek 2008). Meanwhile, soil pH in PR and PSR sites was significantly lower than that in other sites. This is because the increase of organic acid produced by the decomposition of conifer litter from *Pinus massoniana* (Vittori Antisari et al. 2011). The lower pH and SBD in restoration area could in turn accelerated vegetation succession.

Studies have reported that SOM was the basis for other soil properties, and vegetation restoration can promote the SOM input and significantly improve the soil nutrient availability (Bakker et al. 2019; Deng et al. 2017; Jia et al. 2017). In PSR site, SOM was significantly higher than that of PR and NR sites, indicating slow decomposition of litter and absorption of soil nutrient of *Pinus massoniana* needles (Ali et al. 2019; Chen and Cao 2014). The plant root exudation and litter provided carbon sources to soil, and with vegetation succession, the plant root exudation and litter can promote the increase of SOM (Bu et al. 2018; Zhu et al. 2010). Therefore, in the *Pinus massoniana* restoration area, the withered pine needles covered on the surface soil layer caused litter decomposition slowly, and thus *Pinus massoniana* species restoration should mix with broad‐leaved species. Our results also showed that in the vertical soil profile of different study sites, the SOM in the topsoil was significantly higher than that on other soil layers (Figure 5), because of the promotion of nutrient absorption of the dense roots on soil surface (Liu et al. 2020).

This study showed that in the vertical soil profile of different study sites, AK, AN, and AP had a downward trend (Figure 5). This is probably because of soil leaching characteristics and changes in the soil microbial and biomass (Zhao, Liu et al. 2022). In addition, deeper soil obtains limited nutrients from the decomposition of litter, resulting in higher nutrients in topsoil layer (Zhao, Zhao et al. 2022). Our results also showed that the MBC and the MBN in PSR site were significantly higher than that in PR and NR sites (Figure 6). Compared to the *Pinus massoniana* forest, *Pinus massoniana* species mixed with broad‐leaved species forest decomposed litter more effectively, and had higher effectiveness of microbial substrate. Furthermore, there was more supported microbial groups and quantities in the *Pinus massoniana* species mixed with broad‐leaved species forest than *Pinus massoniana* forest, resulted in lower MBC and MBN contents in the PR site.

### 4.3 Coupling Relationship Between Plant Diversity and Soil Properties

Our results indicated that the relationship between plant diversity and soil properties in different layers was significant (Figure 7). The main reason was that soil‐vegetation ecosystem had feedback mechanisms between soil and vegetation, and they can interact with each other (Li et al. 2021). Different vegetation types can affect soil properties through the nutrient release of litter and plant roots (Danise et al. 2021; Yu et al. 2018). SOM was an important factor in plant diversity to sustain the function of plant growth (Kooch, Ehsani, and Akbarinia 2020). In this study, SOM was negatively correlated with the Shannon–Wiener index, Simpson index, and Pielou index. On one hand, under the condition of poor soil nutrients, vegetation improved the growth through the increase of water availability and degree of mineralization, and suitable good water conditions can also accelerate the degradation of understory litter, increase the SOM, and indirectly improve the soil quality (Petersen et al. 2015). On the other, the soil microbial activity can promote SOM accumulation, resulted in increased plant pathogen attack, deterioration plant living environment (Bongiorno et al. 2019; Hagen‐Thorn et al. 2004).

In addition, AP and AN was negatively correlated with plant diversity in different soil depths (Figure 7). The reason is that the increase of plant diversity results in the full utilization and absorption of soil nutrients, and reduces soil phosphorus availability and the leaching loss of *N* in ecosystems (Wang et al. 2010; Zemunik et al. 2015). On the contrary, SOM and AP were positively correlated with Margalef richness index, which is consistent with other studies (Hacker et al. 2015; Zeugin et al. 2010). Firstly, diverse tree species could produce a greater and more diverse litter pool (leaves and roots) in the formation of SOM (Lange et al. 2015; Zeugin et al. 2010). Secondly, the plants functional traits, such as leaf carbon and nitrogen content, can significant improve litter quality and promote the increase of soil *C* and *N* content, and further influence soil decomposition rate and soil nutrients during vegetation restoration process (Münzbergová and Šurinová 2015; van der Putten et al. 2013). Lastly, the increased richness index of vegetation can also mitigate wind and water erosion, avoiding the loss of fine particles and nutrients from the soil (Zhang et al. 2019).

Our results indicated that SBD was positively correlated with the Shannon–Wiener index, Simpson index, and Pielou index but negatively correlated with Margalef index, indicating that SBD was significantly affect the plant diversity. SBD and SWC played an important role in soil hydrological processes (Katherine et al. 2010), and affected the geochemical cycle of plants and microorganisms (Vereecken et al. 2014). Studies have reported that soil pH decrease resulted in the degradation of plant diversity (Xu et al. 2022; Xue, Bezemer, and Berendse 2019). However, although the decrease of soil pH had a negative effect on plant growth, it provided more space for increasing plant diversity (Zhao, Zhao et al. 2022). This indicated that species composition led to changes in community environment, resulted in complex interaction among plant and soil and resources for plant growth, which might diminish the importance of soil properties on plant diversity (Härdtle, von Oheimb, and Westphal 2003; Pérez‐Bejarano et al. 2008). In general, soil provides a better environment for nutrient absorption and growth of plants, thus promoting the colonization and growth of different plant species and improving plant diversity (Gong et al. 2023). Therefore, the plant growth in abandoned coal mine was not only a process of plant adaptation to soil nutrients, but also the interaction of plant growth and soil properties. This result has important guiding significance for the restoration and reconstruction of abandoned coal mine areas vegetation ecosystems. In the process of abandoned coal mine areas vegetation restoration, it is necessary to select not only species that are suitable for the degraded soil environment but also improve soil properties and plant diversity in order to achieve the expected goal of abandoned coal mine areas vegetation restoration.

## 5 Conclusion

The ecological restoration of abandoned mining area should pay attention to the enhancement of soil ecosystem functions and achieving sustainable development. The vegetation configuration of ecological restoration plays a crucial role in accomplishing these goals. Our study showed that (1) there was significant differences in plant diversity and ecological restoration patterns. The PSR site had higher Shannon–Wiener index and Simpson index values than PR site did, and the plant diversity of herb layer was significantly improved than that of the arbor and shrub layers. The plant diversity was slightly higher in the NR and NA site than those of PR and PSR sites. (2) Ecological restoration patterns had a significant effect on the soil properties, and SBD, SWC, SOM, and MBC also significantly affected plant diversity. (3) Identification of dominant plants in revegetation is of great importance for the species selection in abandoned coal mine area. (4) It was recommend that vegetation configuration was of great significance in improving soil properties and increasing plant diversity, vegetation restoration of mixed coniferous with broad‐leaved forests should be paid enough attention to abandoned coal mines ecological restoration.

## Author Contributions

**Wenbo Chen:** conceptualization (lead), funding acquisition (lead), methodology (equal), project administration (lead), supervision (lead), writing – review and editing (equal). **Hao Li:** conceptualization (equal), formal analysis (lead), investigation (lead), methodology (equal), resources (lead), software (lead), validation (equal), visualization (equal), writing – original draft (lead). **Jintai Li:** data curation (equal), formal analysis (equal), investigation (equal), resources (equal), writing – original draft (equal). **Cheng Zhang:** data curation (equal), methodology (equal), validation (equal), writing – review and editing (equal). **Haifen Liang:** investigation (equal), software (equal), visualization (equal), writing – review and editing (equal).

## Conflicts of Interest

The authors declare no conflicts of interest.

## Data Availability

The data presented in this study are available in this manuscript.
